# Fighting Emerging Caspofungin-Resistant *Candida* Species: Mitigating *Fks*1-Mediated Resistance and Enhancing Caspofungin Efficacy by Chitosan

**DOI:** 10.3390/antibiotics13070578

**Published:** 2024-06-22

**Authors:** Aya Tarek, Yasmine H. Tartor, Mohamed N. Hassan, Ioan Pet, Mirela Ahmadi, Adel Abdelkhalek

**Affiliations:** 1 Faculty of Veterinary Medicine, Zagazig University, Zagazig 44511, Egypt; ayatarek020@vet.zu.edu.eg; 2 Department of Microbiology, Faculty of Veterinary Medicine, Zagazig University, Zagazig 44511, Egypt; mnmohamed@zu.edu.eg; 3 Department of Biotechnology, Faculty of Bioengineering of Animal Resources, University of Life Sciences “King Mihai I” from Timisoara, 300645 Timisoara, Romania; mirelaahmadi@usvt.ro; 4 Faculty of Veterinary Medicine, Badr University in Cairo (BUC), Badr City 11829, Egypt; adel.abdelkhalek@buc.edu.eg

**Keywords:** *Candida albicans*, *Candida krusei*, *Candida tropicalis*, caspofungin-resistance, chitosan, *FKS* mutation, histone acetyltransferase genes, cell wall thickness

## Abstract

Invasive candidiasis poses a worldwide threat because of the rising prevalence of antifungal resistance, resulting in higher rates of morbidity and mortality. Additionally, *Candida* species, which are opportunistic infections, have significant medical and economic consequences for immunocompromised individuals. This study explores the antifungal potential of chitosan to mitigate caspofungin resistance in caspofungin-resistant *Candida albicans*, *C. krusei*, and *C. tropicalis* isolates originating from human and animal sources using agar well diffusion, broth microdilution tests, and transmission electron microscope (TEM) analysis of treated *Candida* cells. Reverse transcriptase quantitative polymerase chain reaction (RT-qPCR) was performed to assess the expression of SAGA complex genes (*GCN5* and *ADA2)* and the caspofungin resistance gene (*FKS*) in *Candida* species isolates after chitosan treatment. The highest resistance rate was observed to ketoconazole (80%) followed by clotrimazole (62.7%), fluconazole (60%), terbinafine (58%), itraconazole (57%), miconazole (54.2%), amphotericin B (51.4%), voriconazole (34.28%), and caspofungin (25.7%). Nine unique *FKS* mutations were detected, including S645P (*n* = 3 isolates), S645F, L644F, S645Y, L688M, E663G, and F641S (one isolate in each). The caspofungin minimum inhibitory concentration (MIC) and minimum fungicidal concentration (MFC) values before chitosan treatment ranged from 2 to 8 µg/mL and 4 to 16 µg/mL, respectively. However, the MIC and MFC values were decreased after chitosan treatment (0.0625–1 µg/mL) and (0.125–2 µg/mL), respectively. Caspofungin MIC was significantly decreased (*p* = 0.0007) threefold following chitosan treatment compared with the MIC values before treatment. TEM analysis revealed that 0.5% chitosan disrupted the integrity of the cell surface, causing irregular morphologies and obvious aberrant changes in cell wall thickness in caspofungin-resistant and sensitive *Candida* isolates. The cell wall thickness of untreated isolates was 0.145 μm in caspofungin-resistant isolate and 0.125 μm in sensitive isolate, while it was significantly lower in chitosan-treated isolates, ranging from 0.05 to 0.08 μm when compared with the cell wall thickness of sensitive isolate (0.03 to 0.06 μm). Moreover, RT-qPCR demonstrated a significant (*p* < 0.05) decrease in the expression levels of histone acetyltransferase genes (*GCN5* and *ADA2*) and *FKS* gene of caspofungin-resistant *Candida* species isolates treated with 0.5% chitosan when compared with before treatment (fold change values ranged from 0.001 to 0.0473 for *GCN5*, 1.028 to 4.856 for *ADA2*, and 2.713 to 12.38 for *FKS* gene). A comparison of the expression levels of cell wall-related genes (*ADA2* and *GCN5*) between caspofungin-resistant and -sensitive isolates demonstrated a significant decrease following chitosan treatment (*p* < 0.001). The antifungal potential of chitosan enhances the efficacy of caspofungin against various caspofungin-resistant *Candida* species isolates and prevents the development of further antifungal resistance. The results of this study contribute to the progress in repurposing caspofungin and inform a development strategy to enhance its efficacy, appropriate antifungal activity against *Candida* species, and mitigate resistance. Consequently, chitosan could be used in combination with caspofungin for the treatment of candidiasis.

## 1. Introduction

Life-threatening invasive candidiasis is a global health concern that has become more common in recent years, especially in hospital settings [1]. The most isolated *Candida* species are *Candida albicans* and *C. krusei*, which account for 70–75% of all cases of candidiasis in humans and animals [2]. *Candida* species are opportunistic yeasts that result in infections of the mucosal membranes and deep tissues by colonizing the mucosa of the mouth, esophagus, vagina, and gastrointestinal tract. *Candidiasis* is frequently associated with HIV infection, organ transplantation, cancer, and diabetes, and has a mortality rate of up to 60% [3]. *Candida* species frequently form a biofilm on implanted medical equipment [4]. The necessity for innovative antifungal agents to treat infections caused by these resistant *Candida* species is urgent due to the high mortality rates, high rate of resistance, drug-induced toxicity, and limited number of antifungals.

Azoles, polyenes, and echinocandins are the three major types of antifungals that have been licensed to treat candidiasis [5]. Resistance has emerged with the overuse of antifungals [6].

Echinocandins are lipopeptide antifungals that are structurally distinguished by a cyclic hexapeptide core that is bound to a variety of lipid side chains. It prevents the activity of the glucan synthase enzyme, which is responsible for the production of glucan, a critical polysaccharide component of the fungal cell wall. A plasma membrane-bound catalytic subunit (encoded by the *FKS* gene) and an activating subunit (encoded by the *RHO1* gene) constitute the 1–3 glucan synthase complex [7]. Because echinocandins are more effective and offer a wider antifungal spectrum than azoles against *Candida* spp., they have replaced azoles as the primary antifungal treatment. The emergence of echinocandin-resistant *C. albicans*, *C. krusei*, and *C. glabrata* has been linked to increased use of these antifungal agents [8]. During treatment, echinocandin resistance is typically acquired in various species of *Candida*. Amino acid modifications in hot-spot regions of the *FKS* gene of glucan synthase are responsible for the mechanism of resistance, which diminishes the enzyme’s drug sensitivity [9].

In fact, several studies have shown that the morphological phenotypic change may be regulated epigenetically by chromatin structure modifiers and well-known transcription factors in signaling pathways. Posttranslational histone changes significantly influence the control of this fungus’ pathogenicity, including the yeast-to-hyphae transition, the white-opaque switching, biofilm formation, and drug efflux activity [10]. Epigenetic regulatory systems are now acknowledged as essential regulators of the phenotypic plasticity of *C. albicans*. A rapid and reversible change in the expression of genes and adaptability to adverse environments may result from epigenetic regulation’s detection of environmental changes [11]. Histone acetyltransferases (HATs) and histone deacetylases (HDACs) are opposing enzymes that control histone acetylation and deacetylation, which are important processes in chromatin dynamics [12]. *Candida* can adjust to changes in the environment as well as the stress that is faced when interacting with host cells. This adaptability and pathogenicity both depend on histone deacetylation-mediated chromatin remodeling [13]. The discovery of the *ADA2* (alteration/deficiency in activation gene) was made in *Saccharomyces cerevisiae* [14]. *ADA2* and *GCN5* are components of the *Spt-Ada-Gcn5-*acetyltransferase (SAGA) complex, which is a critical participant in histone acetylation and the regulation of several genes. The histone acetyltransferase (HAT) activity of *Gcn5* enables it to acetylate the N-terminal lysines of histones. *ADA3 (Ngg1)* and *ADA2* are also essential for the histone acetylation mechanism on nucleosomes [15]. Since *C. albicans* have high-similarity homologs of *S. cerevisiae* SAGA complex components, the *ADA2* gene is crucial for histone acetylation [16].

Chitosan, a linear polysaccharide that is naturally occurring, biodegradable, and non-toxic, is produced from deacetylated chitin [17]. Chitosan, with a higher molecular weight, has the potential to produce a polymer barrier on the cell surface, which may prevent the entry of nutrients into the cell. The calcineurin pathway enzyme Crz1p, the stress-response factor Cin5p, and the transcription factor Rlm1p, which is crucial for maintaining cell wall integrity, are activated in chitosan-treated cells [18]. In addition, *S. cerevisiae* treated with chitosan displayed increased resistance to the cell wall-degrading enzyme β-1,3-glucanase, indicating that chitosan-induced plasma membrane stress can be compensated for at the cell wall level through the activation of cell wall integrity transcription pathways [19].

This study investigates for the first time the antifungal potential of chitosan to mitigate caspofungin resistance in *C. albicans*, *C. krusei*, and *C. tropicalis* isolates originated from human and animal sources. Furthermore, the effect of chitosan on the cell architecture and cell wall thickness of caspofungin-resistant and -sensitive clinical isolates as well as its capacity on antagonizing SAGA complex and *FKS* genes expression were examined.

## 2. Results 

### 2.1. Antifungal Susceptibility of Candida Species Isolates

The highest resistance rate was observed for KTZ (80%), followed by CLT (62.7%), FLZ (60%), TERB (58%), ITC (57%), MCZ (54.2%), AmB (51.4%), VRZ (34.28%), and CAS (25.7%) (Table 1). The mean of the inhibition zone diameters was observed for AmB (7.54 ± 1.22 mm), CLT (9.06 ± 1.25 mm), ITC (8.84 ± 1.47 mm), FLZ (11.56 ± 1.04 mm), KTZ (9.40 ± 1.57 mm), MCZ (7.26 ± 1.24 mm), TERB (6.74 ± 1.1 mm), VRC (8.67 ± 1.32 mm), and CAS (11.74 ± 1.16 mm).

*C. albicans* isolates were resistant to KTZ (40%), followed by ITC and CLT (28.5%, each), TERB (25.7%), AmB and MCZ (22.8%, each), FLZ (20%), and CAS (11.43%). Different resistance rates were observed in *C. krusei* isolates as the highest resistance was found for FLZ and KTZ (40%, each), then CLT and TERB (34.2%, each), MCZ (31.4%), AmB and ITC (28.5%), and CAS (11.43%) (Table 1).

Correlation between different antifungals revealed that there were high positive correlation coefficients between the resistance to CLT and KTZ (r = 0.56) and FLZ (r = 0.11). Also, high coefficients were detected between CAS resistance and CLT (r = 0.31) and VRC (r = 0.16) (Supplementary Figure S1).

### 2.2. Detection of Mutations in FKS Genes

Caspofungin resistance in *Candida* species is due to mutations in “hot spot” regions of *FKS*-encoded subunits of glucan synthase, which decrease the sensitivity of the enzyme to the drug, resulting in higher MIC values. Nine isolates with CAS MICs at the intermediate or resistance range (Table 1) were screened for mutations in *FKS*1. Nine unique mutations were detected in *FKS*1 HS1 (Table 2), including S645P (*n* = 3 isolates), S645F, L644F, S645Y, L688M, E663G, and F641S (one isolate for each amino acid change). No mutations were detected in *FKS1* HS2.

### 2.3. Antifungal Activity of Chitosan

The antifungal potential of chitosan against caspofungin-sensitive and -resistant isolates was assessed by the agar-well diffusion assay and broth micro-dilution test. The antifungal activity of chitosan was concentration-dependent (Table S2 and Supplementary Figure S2) as there was a high significant difference (*p* < 0.0001) between inhibition zone diameters of *Candida* species when tested with different concentrations of chitosan, with 0.5% being the most effective one. At 0.5% chitosan, the inhibition zone diameter (IZD) ranged from 10 to 23 mm (the mean value of IZD was 13.485 ± 1.27 mm). At 0.25% concentration, the IZD ranged from 7 to 15 mm (mean value was 8.02 ± 1.12 mm). At 0.125% concentration, the IZD ranged from 7 to 14 mm (mean value was 6.13 ± 1.08), and at 0.0625% concentration, the highest IZD was 10 mm (mean value was 3.26 ± 0.93). After chitosan treatment, the isolates grew as uniform, relatively small colonies when compared with the colony morphology before chitosan treatment.

### 2.4. Chitosan Increases the Susceptibility of Candida Species to Caspofungin

To further confirm the screening results, the caspofungin-resistant and -sensitive isolates were tested for their sensitivity to different concentrations of chitosan (0.5, 0.25, 0.125, and 0.0625) and then for their susceptibility to caspofungin. After treatment with different concentrations of chitosan, caspofungin-resistant isolates showed an IZD ranging from 0.8 to 20 mm when compared with the IZD for caspofungin before chitosan treatment (Table S3). Caspofungin-sensitive isolates showed a higher IZD ranging from 10 to 25 mm when compared with its zone diameters before chitosan treatment. Caspofungin MIC and MFC values ranged from 2 to 8 µg/mL and 4–16 µg/mL, respectively (Table 2). However, the MIC and MFC values after chitosan treatment became 0.0625–1 µg/mL and 0.125–2 µg/mL, respectively. The MIC of caspofungin was significantly decreased (*p* = 0.0007) threefold following chitosan treatment compared with the MIC values before treatment.

### 2.5. Chitosan-Induced Changes in Cell Wall Thickness of Caspofungin-Resistant Candida Species

**Figure 1:**
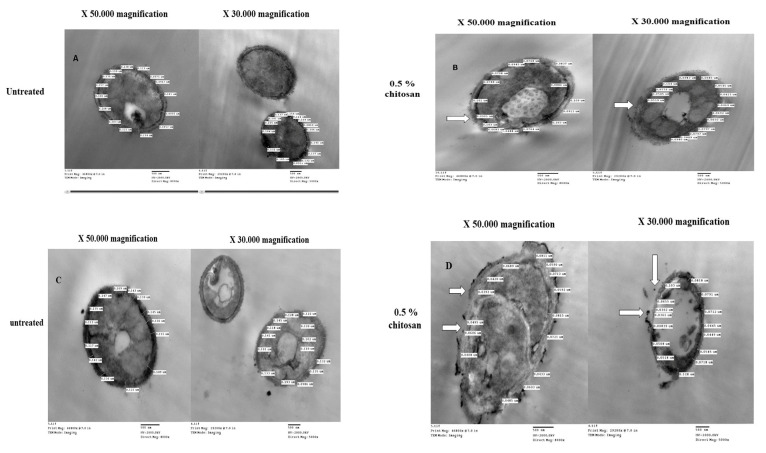
TEM images for individual cells of caspofungin-resistant isolates (**A**,**B**) and caspofungin-sensitive isolates (**C**,**D**) that were obtained at ‘’50,000’’ ‘’X’’ and ‘’30,000’’ ‘’X’’ magnifications, randomly selected before chitosan treatment and their cell wall thickness was measured. Twenty sites around the circumference of each selected cell were measured. Individual cells were randomly selected after chitosan (0.5%) treatment and their cell wall thickness was measured. The integrity of the *Candida* cell wall and cell membrane was disrupted and exhibited slightly irregular cell morphologies after chitosan treatment. White arrows identify disruption of *C. albicans* cell walls after chitosan 0.5% treatment.

**Figure 2:**
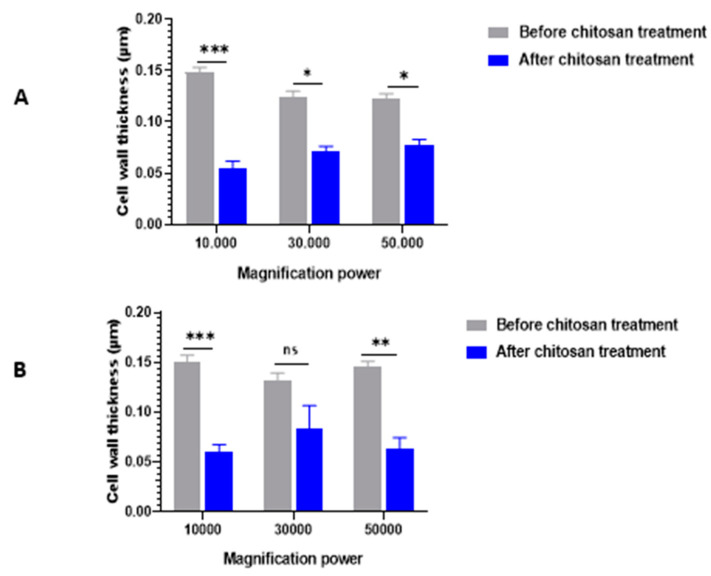
(**A**) Cell wall thickness of caspofungin-resistant isolates was significantly reduced after 0.5% chitosan treatment compared with those before-treatment at magnification powers ‘’10,000’’, ‘’30,000’’and ‘’50,000’’. The values are the means ± standard errors (SE). *** *p* < 0.001; * *p* < 0.05. Statistical significance was determined using the Student’s *t*-test. (**B**) The cell wall thickness of caspofungin-sensitive isolates was significantly reduced after chitosan treatment compared with before treatment at magnification powers ‘’10,000’’ and ‘’50,000’’. However, non-significant differences were observed at magnification power ‘’30,000”. The values are the means ± SE. *** *p* < 0.001; ** *p* < 0.01; ns *p* > 0.05. Statistical significance was determined using the Student’s *t*-test.

TEM examination of control (untreated) *C. albicans* cells showed typical morphology of *Candida* with a uniform density, typically structured nucleus, and cytoplasm with several elements of an endomembrane system enveloped by a regular, intact cell wall (Figure 1A,B). After 24 h treatment with 0.5% chitosan, the cells exhibited notable alterations in the cell membrane and the cell wall, causing irregular morphologies that were occasionally visible, especially in sensitive isolates. Chitosan principally interacts with the yeast cell wall, causing severe swelling and asymmetric rough shapes, and subsequent cell wall lysis. More significantly, there was an alteration in the morphology of the cells; the integrity of the cell walls was unclear, and it was unclear where the cell wall and cell membrane separated. Comparison between cell wall thickness and cell membrane revealed that *C. albicans* cells exhibited an extremely thin cell wall for resistant (Figure 1A,B) and sensitive isolates (Figure 1C,D). Three cell wall thicknesses were measured in twenty sites around the circumference of each cell. The quantitative analysis revealed that the untreated isolate had a cell wall thickness of 0.145 μm, measured between the plasma membrane and the cell wall. In contrast, the cell wall thickness of the chitosan-treated isolate was significantly lower (*p* < 0.05), ranging from 0.05 to 0.08 (Figure 2).

Interestingly, chitosan-treated cells for both isolates appear transparent with evidence of a loss of cell morphology, potentially resulting from an efflux of intracellular material following penetration of the cell by chitosan.

### 2.6. Real-Time RT-PCR for Gene Expression Analysis

**Figure 3:**
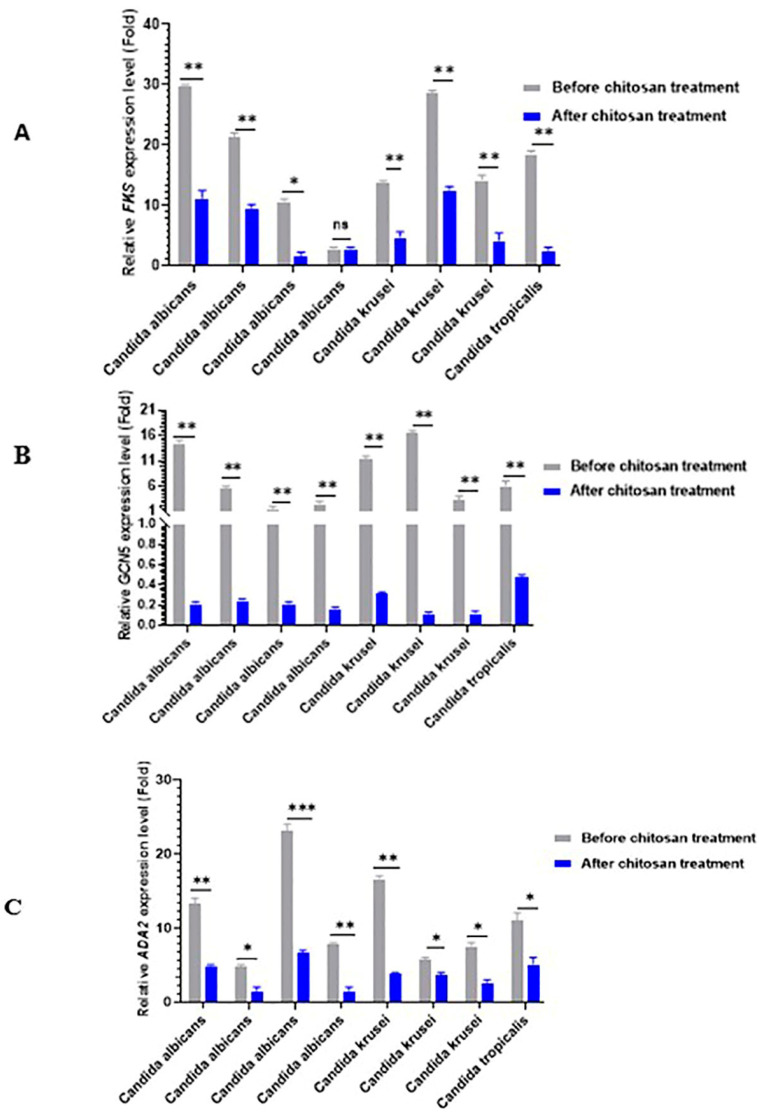
The expression levels of cell wall-related genes. (**A**) *FKS*, (**B**) *GCN5*, and (**C**) *ADA2* genes in caspofungin-resistant isolates. All examined isolates showed a significant decrease following treatment with chitosan 0.5% compared with the control group. The mean values ± standard errors (SE) are provided. * *p* < 0.05; ** *p* < 0.001, *** *p* < 0.001, and ns non-significant. Statistical significance was determined using the Student’s *t*-test.

**Figure 4:**
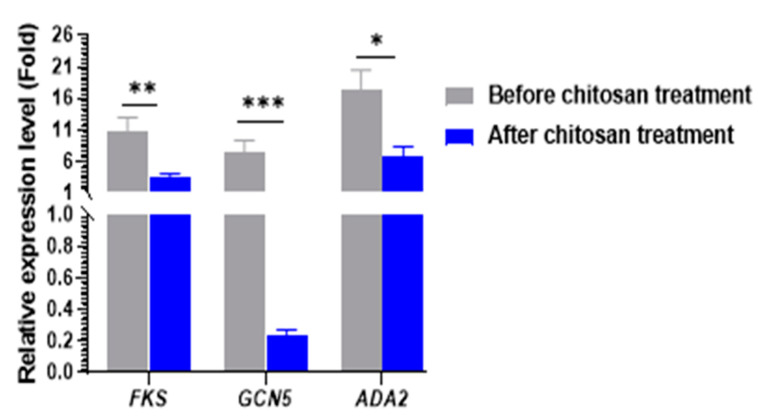
The expression levels of cell wall-related genes in *C. albicans, C. krusei*, and *C. tropicalis*, specifically *FKs*, *GCN5*, and *ADA2* genes showed a significant decrease following treatment with chitosan when compared with the untreated isolates. The mean values ± standard errors (SE) are provided. * *p* < 0.05; ** *p* < 0.001, *** *p* < 0.001. Statistical significance was determined using the Student’s *t*-test. The expression values were compared between caspofungin-resistant and -sensitive isolates treated with and without chitosan. The expression was normalized to the *ACT1* gene.

To further confirm the inhibitory effects of chitosan on histone acetyltransferase genes (*GCN5* and *ADA2*) and caspofungin resistance gene (*FKS*) in *Candida* species isolates, the transcript levels of *GCN5*, *ADA2*, and *FKS* genes were determined by reverse transcriptase quantitative PCR (RT-qPCR). *Candida* species isolates showed low transcript levels of the histone acetyltransferase genes, *GCN5* (fold change range = 0.001–0.0473 and *ADA2* (fold change range = 1.028–4.856), and *FKS* gene (fold change range = 2.713–12.38 after chitosan treatments when compared with the untreated isolates. Notable significant differences were observed in the expression levels of the two histone acetyltransferase genes (*GCN5-ADA2* SAGA complex (*p* < 0.05) in caspofungin- resistant isolates (Figure 3A–C). As presented in Figure 4, comparison of the expression levels of cell wall-related genes between caspofungin-resistant and -sensitive isolates treated with and without chitosan revealed a significant decrease following chitosan treatment (*p* < 0.001). The expression level was normalized to the *ACT1* gene.

## 3. Discussion

Invasive fungal infections pose a substantial infection risk for individuals with underlying immunosuppression. Serious fungal diseases are becoming increasingly prevalent, resulting in the deaths of over 1.5 million individuals annually. This issue is further complicated by the emergence of drug-resistant and/or multidrug-resistant species, particularly in *Candida* species [20,21]. The classification of echinocandins as fungicidal or fungistatic is dependent upon the species and strain of the fungus. Anidulafungin, micafungin, and caspofungin were the most successful echinocandins. These drugs are suitable for use as first-line treatments due to their potent activity, wide range, high safety, excellent pharmacokinetics and pharmacodynamics, and minimal drug-drug interactions, particularly in the context of nosocomial candidemia [22]. Recently, there has been a significant increase in the focus on the bioactivity of natural compounds and their practical applications in pharmaceutical research. Despite the fact that the majority of antibacterial compounds extracted from marine sources have not been sufficiently efficacious to compete with conventional antibiotics derived from microbes, the majority of marine creatures have not yet been investigated to identify valuable antibiotics [21,22,23]. The aim of this study was to investigate the role of chitosan as a biological factor for the mitigation of caspofungin resistance and enhancing its efficacy against resistant *C. albicans*, *C. krusei*, and *C. tropicalis* isolated from human and animal samples and its effect on the *FKS* gene and histone acetyltransferase genes.

*Candida* species frequently exhibit resistance to a wide variety of antifungals [4]. In this study, *C. albicans* isolates were resistant to ketoconazole (40%) followed by itraconazole and clotrimazole (28.5%, each), terbinafine (25.7%), amphotericin B and miconazole (22.8%, each), fluconazole (20%), and caspofungin (11.43%). However, it has been reported that *C. albicans* isolates exhibited high resistance rate to itraconazole (64.3%), clotrimazole (50%), fluconazole (46.4%), and ketoconazole (39.3%) [4]. Moreover, Badiee et al. [24] declared that *C. albicans* strains were resistant to itraconazole (33.7%), fluconazole (10.7%), ketoconazole (9.4%), amphotericin B (7%), and caspofungin (1.8%). Nevertheless, *C. krusei* strains were resistant to fluconazole (66.6%), amphotericin B (3.1%), ketoconazole (10.4%), itraconazole (86.5%), and caspofungin (4.2%). Here, different resistance rates were observed in *C. krusei* isolates and the highest resistance was found for fluconazole and ketoconazole (40%, each), then clotrimazole and terbinafine (34.2%, each), miconazole (31.4%), amphotericin B and itraconazole (28.5%), and caspofungin (11.43%). Resistance rates are dependent upon the species, antifungal agents, and the duration of fungistatic drug use, which may result in the pathogenic yeasts developing resistance, thereby diminishing the drug’s efficacy.

At present, the only clinically significant acquired resistance of *Candida* species to echinocandins has been linked to single amino acid substitutions that result from mutations within conserved (“hot spot”) sequences of the *FKS1* and *FKS2* genes in *C. glabrata*, which encode for glucan synthase. *FKS* mutations were the sole significant predictor of clinical echinocandin treatment failure in multivariate analyses instead of echinocandin MIC. For this reason, the most precise method of classifying echinocandin-resistant isolates may be the molecular detection of *FKS* mutations. Currently, there are no commercially available assays for *FKS* genotyping, which restricts molecular testing of resistance in reference laboratories [25]. Our findings correspond with the findings conducted by Coste et al. [26] that the point mutation S645P is predominant in *C. albicans* strains. All *FKS*-mutant isolates exhibited caspofungin MICs that were interpreted as non-susceptible according to the CLSI breakpoints and were similar to those reported in France from 2004 to 2010 that eight caspofungin-resistant *C. albicans* have different types of mutations in the HS1 region, while two *C. krusei* isolates had two different mutations in the HS1 region [27].

We investigated the antifungal properties of chitosan against all isolates of *Candida* species. There was a range of 10 to 23 mm in the IZD at 0.5% chitosan. At a concentration of 0.25%, the IZD varied from 7 to 15 mm. This was in accordance with the study conducted by Yadav et al., which determined that the IZD of 0.5% chitosan against *C. albicans* was 21 mm [28]. Nevertheless, 15 mm IZD has been achieved for *Candida* species using 1.5% low molecular weight (LMW) chitosan [29].

Chitosan converts caspofungin-resistant isolates to caspofungin-sensitive isolates (IZD of 10 to 25 mm). Prior to chitosan treatment, the caspofungin MIC and MFC values were 2–8 µg/mL and 4–16 µg/mL, respectively. Nevertheless, the caspofungin MIC and MFC values decreased following chitosan treatment (range: 0.0625 to 1 µg/mL) and (0.125 to 2 µg/mL), respectively. Our results were inconsistent with the findings of Abdelatti et al. [30] that the effects of chitosan-amphotericin B and chitosan-caspofungin as combinations against *C. albicans* and *C. tropicalis* isolates were indifferent. Nevertheless, the inhibitory effects of the combinations of chitosan-amphotericin B and chitosan-caspofungin were not observed, even though *C. albicans* SC5314 was highly susceptible to amphotericin B (MIC: 1.0 μg/mL), fluconazole (0.125 μg/mL), and caspofungin (0.25 μg/mL).

The antibacterial action of chitosan is closely linked to the degree of deacetylation and pH [31]. Particularly, the antimicrobial activity of chitosan is enhanced by increased deacetylation. Increased antimicrobial activity is associated with lower pH values [32]. Under acidic conditions, the amine groups of chitosan are protonated, resulting in the chitosan molecules becoming polycationic. The biological activity arises from the positive charge of protonated amine groups. These groups enable the interaction between protonated chitosan molecules and negatively charged proteins, fatty acids, lipids, and nucleic acids present in the fungal cell. Consequently, this process causes the breakdown of fungal cell membranes, confinement of cell components, and the resulting antifungal activity. Chitosan exhibits activity exclusively in an acidic environment [33]. Nevertheless, it has been noted that the restricted level of activity observed at pH 6 may be due to the low solubility [34]. The activity of chitosan against *Staphylococcus aureus* isolates was equal at pH 5.5 and 7.2 (MIC = 256 μg/mL). Therefore, when the negatively charged microbial cell surface interacts with the positively charged chitosan, an inhibitory effect may result from the disruption of the anion-cation balance [35]. Therefore, the antifungal properties are associated with the interaction of chitosan with the cell wall or cell membrane. However, the molecular weight (MW) and level of deacetylation of chitosan, the pH of the solvent, and the type of fungus being targeted are all strongly correlated with the MICs of chitosan against fungi [36].

Several studies [37,38] declared that the antifungal activity of chitosan can be influenced by its MW. Variations in MW can impact the properties of chitosan in two ways: First, living cells are susceptible to the penetration of LMW chitosan, which can block several enzymes and impair protein synthesis, hence interfering with mRNA synthesis. Second, there is an increased adsorption of high molecular weight (HMW) chitosan on cell walls, which causes cell walls to be covered, the membrane to weaken and break, and cells to leak. Furthermore, chitosan chelates essential minerals and metals to stop the growth of bacteria [37] and fungi [39]. Electrostatic interactions between the protonated NH^3+^ groups and the negative residues are responsible for the mode of action [40], presumably by competing with Ca^2+^ for electronegative regions on the surface membrane [41]. Furthermore, the chitosan susceptibility may be positively correlated with the unsaturated fatty acid content of the cell membrane. This is due to the fact that a higher content of unsaturated fatty acids results in improved membrane fluidity, which in turn leads to a more negative charge on the cell membrane [42]. According to the screening results, the chitosan employed in this investigation possessed prospective intracellular action in addition to extracellular interaction capabilities. Some fungal pathogens, including *Cryptococcus neoformans*, employ chitosan to preserve the integrity of their cell walls during the vegetative phase [43].

Indeed, TEM analysis verified that chitosan, albeit at a concentration of 0.5%, induced obvious aberrant changes in cell wall thickness in caspofungin-resistant and sensitive isolates. The quantitative results indicated that the untreated isolates had a cell wall thickness of 0.145 μm for the resistant isolate and 0.125 μm for the sensitive isolate, as measured between the plasma membrane and the cell wall. In contrast, the cell wall thickness of chitosan-treated isolates was significantly lower, ranging from 0.07 to 0.08 and 0.05 to 0.06 for the resistant isolate, respectively, when compared with the cell wall thickness of the sensitive isolate, which was 0.03 to 0.04 μm and 0.05 to 0.06 μm. This is in accordance with the results reported by Shih et al. that the cell wall thickness of the untreated strain was 0.125 μm, whereas the chitosan-treated strains had a substantially lower cell wall thickness, ranging from 0.04 to 0.05 μm and 0.06 to 0.07 μm, respectively [39]. This may be attributed to the antimicrobial activity of chitosan, which targets the cell surface, suggesting that chitosan is a potential alternative approach to antifungal agents against resistant fungi [44]. The cell wall of susceptible isolates is widened, in contrast to that of isolates with reduced susceptibility. This was linked to elevated levels of β-1,3-glucan in the cell wall, with no noticeable modifications in chitin content. The proteomic analysis revealed modifications in the repertoire of proteins involved in cell wall organization and maintenance in drug-resistant strains in comparison to susceptible strains following incubation with caspofungin [45].

In the current study, the RT-qPCR assay was employed to investigate the expressions of the *GCN5*, *ADA2*, and *FKS* genes both before and after chitosan treatment. Compared with the non-treated isolates, the relative expression of *GCN5* and *ADA2* genes was significantly reduced in chitosan-treated isolates. This could be attributed to the inhibition of SAGA complex expression by chitosan, which is one of the biocidal mechanisms of chitosan against *C. albicans*. This hypothesis was additionally confirmed by Shih et al.‘s findings that chitosan 0.2% inhibited *ADA2* expression. Additionally, chitosan induces the repression of the *GCN5* histone acetyltransferase [16]. *Neurospora crassa* genes, such as *gel-1* (involved in glucan elongation) and β-1,3-glucan synthesis (*FKS*), were significantly reduced by chitosan. The downregulation was observed in the presence of caspofungin and at all concentrations of chitosan (10 and 30 μg /mL) [46]. The results suggest that the chitosan treatment of *Candida* cells either reduces the levels of chitin and β-glucan or modifies the ultrastructure of the cell wall and cell membrane by inhibiting the expression of SAGA complex components. As a result, tangible evidence to substantiate our hypothesis can be obtained by examining the composition and organization of the cell wall in both chitosan-treated and untreated cells. Additionally, it is probable that the outcome arises from a combination of indirect mechanisms, where multiple signaling pathways are necessary for the response to chitosan. This can result in a decrease in cell wall strength and changes in gene expression [47].

In budding yeast and *Candida* species, the *ADA2-ADA3-GCN5* SAGA complex is sufficient for the induction or repression of the expression of specific genes, resulting in robust HAT activity [48]. In *C. albicans*, the *ADA2* gene is crucial for histone acetylation; strains with *ADA*2 mutations had less H3K9 acetylation. The recruitment of *ADA2*, its ability to bind to 200 gene promoters, and its role in mediating the expression of several genes, including those involved in glycolysis, pyruvate metabolism, oxidative stress, drug responses, and cell wall responses, were further confirmed by chromatin immunoprecipitation assays [49]. *Gcn5* may acetylate N-terminal lysines on histones and demonstrates HAT activity. Moreover, *Ada3 (Ngg1)* and *ADA2* are required for the histone acetylation process on nucleosomes [50].

Consequently, the reversal of caspofungin resistance to sensitivity is most likely the result of altered cell wall composition and dysregulated glucan synthesis enzymes, such as *FKS1*, *FKS2*, and *FKS3*, as *GCN5*-mediated regulation affects signaling pathways that are required for the regulation of morphogenetic changes, including filamentation and virulence [51]. Conversely, previous research indicated that 3 mg/mL of chitosan, in both solution and nanoparticle form, are required to inhibit 90% of *C. albicans*, *Aspergillus niger*, and *Fusarium solani*. The natural antifungal activity of chitosan was, therefore, less potent than that of synthetic antifungal agents [52]. Thus, chitosan in combination with caspofungin may increase its antifungal activity. Moreover, chitosan could also inhibit the *FKS* gene, which leads to a rise in the efficacy of antifungals against resistant isolates.

## 4. Materials and Methods

### 4.1. Isolates

Thirty-five clinical isolates were included in this study. The isolates were recovered from different human and animal samples between 2022 and 2023. Twenty-five clinical cases were admitted at the clinics of different hospitals. From the clinical samples tested and considered in this study, fourteen cases were from human urine samples, three were from sputum, five were from onychomycosis cases, and three were from vaginitis cases in pregnant women. In addition, ten samples from diarrhea in calves were included. Sabouraud dextrose agar with chloramphenicol (SDA, Oxoid Ltd., Cambridge, UK) was used for culture. The isolates were identified based on their characteristics on HiCrome *Candida* differential agar medium (Himedia Laboratories, Mumbai, India), micromorphology on rice agar with Tween 80, and the germ tube test. Matrix-assisted laser desorption/ionization- time of flight mass spectrometry (MALDI-TOF MS)-based identification of all isolates to the species level was performed according to Bruker Daltonics (Biotyper RTC software, v 3.0 (Bruker Daltonics, Bremen, Germany) using the ethanol (EtOH)/formic acid (FA) extraction protocol [53].

### 4.2. Antifungal Susceptibility Testing

Disk diffusion testing was performed according to the Clinical and Laboratory Standards Institute (CLSI) protocol, as outlined in the CLSI M44-Ed3 [54]. The clinical *Candida* species isolates were tested for their susceptibility to various antifungal agents including caspofungin (CAS; 5 μg), amphotericin B (AmB; 10 μg), fluconazole (FLZ; 25 μg), voriconazole (VRZ; 1 μg), terbinafine (TERB; 30 mg), itraconazole (ITC; 10 μg), ketoconazole (KTZ; 15 μg), miconazole (MCZ; 10 mg), and clotrimazole (CLT; 10 μg). All disks were purchased from Sigma-Aldrich Quimica, Madrid, Spain. The suspension of each isolate was adjusted to McFarland No. 0.5 and plated on Mueller-Hinton agar (SSI Diagnostica, Hillerd, Denmark) enriched with 2% dextrose and 0.5 μg/mL methylene blue. The plates were incubated at 37 °C for 24 h. The inhibition zones diameters (IZD) were measured and analyzed according to the CLSI M44-ED3 guidelines.

### 4.3. Determination of Minimum Inhibitory Concentrations

The minimum inhibitory concentration (MIC) of CAS was determined using the broth microdilution method according to the CLSI M27M44S guidelines [55] standard. CAS (Cancidas^®^, Merck & Co. Inc., Rahway, NJ, USA) was dissolved in sterile water and prepared at a concentration range of 0.015 to 4 μg/mL. The isolates were cultured on SDA at 35 °C for 24 h, then the inoculum (1–5 × 10^6^ cells/mL) was prepared in 5 mL of sterile water using the 0.5 McFarland standard. Afterwards, a 1:50 dilution, followed by a final dilution of 1:20 in RPMI 1640 supplemented with 3-(N-morpholino) propanesulfonic acid (MOPS) (Sigma-Aldrich, St. Louis, MO, USA) were performed to reach a final concentration of 1–5 × 10^3^ cells/mL. The inoculum (100 µL) was transferred to 100 µL of medium in a 96-well microplate. The CAS was twofold serially diluted to a concentration range of 0.016 to 8 μg/mL. Growth control (wells containing RPMI-MOPS and inoculum) and sterility control (wells containing RPMI-MOPS), and quality control strains were included in the test. Plates were then incubated at 35 °C for 24 h. The MIC of CAS was reported as the lowest concentration that inhibits 50% of the growth compared with the growth controls. Twenty microliters from each well were plated on SDA and incubated at 35 °C for 24 h to confirm the colony counts. The MFC was defined as the lowest concentration that prevented visible growth according to CLSI document M27-A2 [56]. To ensure rigorous and reproducible data, each experiment was performed in triplicate.

### 4.4. Sequencing of FKs Gene for Detection of Mutations

Genomic DNA from fresh cultures of caspofungin-resistant isolates was extracted using a Qiagen DNA kit (QIAGEN, Hilden, Germany) following the manufacturer’s instructions. The oligonucleotide primers listed in Supplementary Table S1 were used for the amplification of the *FKS* gene and sequencing [57]. The NanoDrop™ 2000/2000c spectrophotometers (Thermo Fisher Scientific, Waltham, MA, USA) were employed to quantify the DNA concentrations. A 25 µL reaction mixture containing 12.5 µL of EmeraldAmp Max PCR Master Mix (Takara, Shigi-no-higashi, Joto-ku, Osaka, Japan), 1 µL of each primer (20 pmol; Biobasic, Toronto, ON, Canada), 4.5 µL of nuclease-free water, and 6 µL of DNA template was used to conduct PCR assays in a T3 thermal cycler (Thermo Scientific, Waltham, MA, USA). The cycling conditions were as follows: initial denaturation at 98 °C for 30 s, followed by 30 cycles of 98 °C for 10 s, 51 °C for 10 s, 72 °C for 30 s, and a final extension step at 72 °C for 5 min. PCR products were electrophoresed on a 1.5% agarose gel (Applichem GmbH, Armstadt, Germany), and an Alpha Innotech gel documentation system (Biometra GmbH, Göttingen, Germany) was used for imaging the gel.

The PCR products were purified by using PureLink PCR Purification kits (ThermoFisher Scientific, Waltham, MA, USA) according to the manufacturer’s instructions. A BigDye Terminator v3.1 cycle sequencing kit (Applied Biosystems, Tokyo, Japan) was used for DNA sequencing in the ABI 3100 Genetic Analyzer (Applied Biosystems, Foster City, CA, USA). Nucleotide sequences were compared with the available sequences in the NCBI database using the Basic Local Alignment Search Tool (www.ncbi.nlm.nih.gov/BLAST/, accessed on 13 April 2024). The amino acid sequences were deduced with the standard genetic code using the Expert Protein Analysis System Translate Tool (ExPASy; https://www.expasy.org/, accessed on 13 April 2024). For detection of mutation, nucleotide and amino acid sequences were aligned with the reference strains *C. albicans SC* 5341, *C. krusei* ATCC 6258, and *C. tropicalis* ATCC 750 using the BioEdit program 7.2 (BioEdit.exe) (Ibis Therapeutics, Carlsbad, CA, USA). DNA sequences were deposited into the GenBank database (accession numbers PP663620–PP663628).

### 4.5. Screening of Antifungal Activity of Chitosan against Candida species Isolates

The antifungal activity of chitosan was determined by the well diffusion test [58]. Chitosan with molecular weight (MW) 50,000–190,000 and 75–85% deacetylation (Sigma Aldrich, St. Louis, MO, USA) was dissolved in 1% acetic acid (Sigma-Aldrich Quimica, Madrid, Spain) to obtain different concentrations (0.5, 0.25, 0.125, and 0.0625). Yeast suspensions were prepared by suspending single colonies in sterile 0.85% saline. The turbidity of each suspension was adjusted to 1.5 × 10^6^ CFU/mL. The suspensions were evenly spread and inoculated on SDA plates and SDA agar containing 1% acetic acid (solvent control group). Six-millimeter diameter wells were made in the agar plate using a sterile cork borer and filled with 100 µL of each concentration of chitosan (0.5, 0.25, 0.125, and 0.0625). The plates were left for 2 h at 4 °C for diffusion and then incubated at 37 °C for 24 h. Growth of the isolates was observed, and the IZD was recorded. The experiment was repeated three times, and the mean values were calculated.

### 4.6. Antifungal Susceptibility Testing for Caspofungin after Chitosan Treatment

Following chitosan treatment, disk diffusion testing was performed using Mueller-Hinton agar (SSI Diagnostica, Hillerd, Denmark) supplemented with 2% dextrose and 0.5 μg/mL methylene blue according to the CLSI M44-Ed3 protocol [54]. The IZD were measured after 24 h of incubation, and the results were recorded. *Candida* species isolates were tested twice for CAS MIC (before and after chitosan treatment).

To confirm the inhibitory influence of chitosan on various *Candida* species isolates, the broth microdilution test was conducted for each isolate following chitosan treatment. The MIC was established by preparing four concentrations of chitosan (0.5%, 0.25%, 0.125%, and 0.0625%) in 1% acetic acid and adding them to RPMI-1640. The plates were then incubated for 24 h before being washed with phosphate buffer saline (PBS) twice and plated onto SDA. Afterward, fresh culture colonies were selected, and the CAS MIC was determined according to the M27M44S protocol of the CLSI guidelines [55].

### 4.7. Transmission Electron Microscopy Analysis

Both caspofungin resistant- and -sensitive isolates were cultured in RPMI 1640 medium as [untreated group] and RPMI 1640 medium supplemented with 0.5% chitosan as a [treated group] at 30 °C for 20 min. They were then rinsed three times with sterile water. The cleaned samples were first fixed with 2.5% glutaraldehyde (Sangon Biotech Co. Ltd., Shanghai, China) for 80 min at room temperature, followed by three 15 min washes with 0.1 M PBS. After fixation for 1 h at room temperature with 1% osmium tetroxide (Sigma-Aldrich Quimica, Madrid, Spain), the samples were washed three times for 15 min using a 0.1 M phosphate buffer. Afterward, the samples were dehydrated by immersing them in ethanol at progressively higher concentrations (30%, 50%, 70%, 90%, and 100%) for 15 min each. Additionally, they were subjected to additional dehydration twice in 100% acetone for 30 min each. The samples were embedded for 4 h in different ratios of acetone Spurr’s resin. This was followed by embedding in pure Spurr’s resin for 48 h at 70 °C [59]. Thin slices of 50–70 nm thickness were cut from the blocks of samples using an ultramicrotome. Images at magnifications of 10,000, 30,000, and 50,000 were acquired using JEOL, JEM-1400 (Tokyo, Japan) to assess the thickness of cell walls. Digital microscopy software e J 1.45s software1493 was used to quantify the measures of the cell wall thickness of *Candida* cells.

### 4.8. Quantitative Reverse Transcription Polymerase Chain Reaction

Two hundred microliters of Sabouraud broth containing *Candida* cells, which had been grown overnight, was transferred to 10 mL of fresh Sabouraud broth. The cells were then centrifuged at 3000 rpm for 10 min, after which they were washed with sterile water three times. Subsequently, the cells were treated with RPMI 1640 medium and RPMI 1640 medium supplemented with 0.5% chitosan at 30 °C for 20 min. Following this treatment, the cells were once again centrifuged at 3000 rpm for 10 min and washed with sterile water three times [60]. Total RNAs were then extracted using a RNeasy Mini Kit (Qiagen, Germany) following the manufacturer’s instructions. The whole procedure was performed under DNAse-free conditions to avoid DNA contamination with 5 μL of Turbo DNase buffer and 1 μL of Turbo DNase enzyme (Thermo Fisher Scientific, Waltham, MA, USA). The real-time PCR mixture (25 µL) consisted of 3 µL of template RNA, 0.25 µL of RevertAid Reverse Transcriptase (Thermo Fisher Scientific, Waltham, MA, USA), 12.5 µL of 2× QuantiTect SYBR Green PCR Master Mix (Invitrogen, Paisley, UK), 0.5 µL of forward and reverse primers (20 pmol) of each gene (*ADA2*, *GCN5*, and *FKS1*) (Table S1), and 8.25 microliters of nuclease-free water. Three separate sets of samples from each strain for each treatment were examined three times using a Stratagene MX3005P qPCR System. The cycling conditions were as follows: 95 °C for 20 s, followed by 40 cycles of 95 °C for 1 s and 60 °C for 20 s. The data were processed using Stratagene MX3005P Mxpro Mx3005p V4 10 software (Stratagene, Amsterdam, The Netherlands). The normalization of gene expression was performed using the *ACT1* housekeeping gene, following the 2^−ΔΔCT^ method [61]. The fold changes were determined as the mean normalized expression of treated strains relative to the mean normalized expression of the untreated one.

### 4.9. Data Analysis

Data were processed in Microsoft Excel (Microsoft Corporation, Redmond, WA, USA) provided by the SPSS software V18 (Chicago. IL, USA). A Shapiro-Wilk test was used to assess normality, as outlined by Razali and Wah [62]. The Student’s *t*-test was used for testing the difference between the pre- and post-chitosan treatments on the cell wall-related genes and cell wall thickness (SAS Institute Inc., Cary, NC, USA) [63], setting the level of significance at α = 0.05. The correlation matrix between different antifungals was fitted according to the Corr procedure. The differences between different treatments on IZD were examined according to one-way Anova. Results were expressed as means ± SE. Figures were fitted by the GraphPad Prism software 9.0 (GraphPad, Boston, MA, USA).

## 5. Conclusions

The antifungal potential of chitosan enhances the efficacy of caspofungin against various caspofungin-resistant *Candida* species isolates and prevents the development of further antifungal resistance. The results of this study contribute to the progress in repurposing caspofungin and to an informed development strategy to enhance its efficacy, ensure appropriate antifungal activity against *Candida* species, and mitigate resistance. Consequently, chitosan could be used in combination with caspofungin for the treatment of candidiasis. Future research may examine the efficacy of combinational therapy by combining chitosan with caspofungin in a clinical trial or with other combinations that could result in novel drug therapies against drug-resistant *Candida* species in medical settings.

## Figures and Tables

**Table 1:** Antifungal resistance pattern of *Candida* species isolates and caspofungin minimum inhibitory concentration values before and after chitosan treatment.

Isolate No.	Species	Source	Resistance Pattern	Caspofungin MIC (µg/mL)	Caspofungin MIC after 0.5% Chitosan Treatment (µg/mL)
C1	*C. albicans*	Calf, Diarrhea	FLZ, CLT, TERB, KTZ, ITC, CAS	2	0.25
C2	*C. krusei*	Urine, Human	FLZ, CLT, KTZ, ITC, TERB, MCZ VRZ, CAS	4	0.5
C3	*C. krusei*	Onychomycosis, Human	FLZ, CLT, ITC, AMB, MCZ, VRZ, TERB, CAS	8	0.25
C4	*C. albicans*	Urine, Human	FLZ, CLT, AMB, TERB, KTZ, ITC, MCZ, VRZ, CAS	2	1
C5	*C. krusei*	Urine, Human	FLZ, AMB, ITC, TERB, VRZ, CAS	8	0.125
C6	*C. albicans*	Calf, diarrhea	FLZ, CLT, TERB, KTZ, ITC, VRZ, CAS	2	0.0625
C7	*C. krusei*	Calf, diarrhea	FLZ, CLT, KTZ, CAS	2	0.25
C8	*C. albicans*	Urine, Human	FLZ, CLT, AMB, KTZ, TERB, VRZ, MCZ, CASP	4	0.25
C9	*C. tropicalis*	Sputum, Human	CLT, AMB, TERB, KTZ, ITC, VRZ, MCZ, CAS	8	0.125
C10	*C. albicans*	Urine, Human	FLZ, CLT, KTZ, ITC, VRZ, MCZ	0.25	0.125
C11	*C. albicans*	Onychomycosis, Human	FLZ, CLT, TERB, KTZ, AMB	0.125	0.0625
C12	*C. krusei*	Urine, Human	FLZ, CLT, VRZ, MCZ, AMB, TERB	0.25	0.125
C13	*C. krusei*	Urine, Human	FLZ, CLT, TERB, KTZ, ITC, VRZ, MCZ	0.25	0.125
C14	*C. krusei*	Urine, Human	FLZ, TERB, KTZ, MCZ	0.125	0.0625
C15	*C. krusei*	Onychomycosis, Human	FLZ, CLT, KTZ, TERB, MCZ	0.25	0.125
C16	*C. krusei*	Vaginal swab, Human	FLZ, CLT, KTZ	0.125	0.065
C17	*C. krusei*	Urine, Human	FLZ, CLT, TERB, KTZ, ITC, MCZ	0.25	0.125
C18	*C. albicans*	Calf, diarrhea	CLT, AMB, KTZ, ITC, MCZ	0.0625	0.03125
C19	*C. krusei*	Sputum, Human	AMB, KTZ, MCZ	0.125	0.0625
C20	*C. krusei*	Calf, diarrhea	AMB, ITC, MCZ	0.0625	0.03125
C21	*C. krusei*	Calf, diarrhea	FLZ, AMB, KTZ	0.125	0.03125
C22	*C. krusei*	Human, Urine	FLZ, CLT, TERB, KT, VRZ	0.25	0.03125
C23	*C. albicans*	Urine, Human	CLT, AMB, TERB, KTZ, ITC	0.25	0.0625
C24	*C. albicans*	Vaginal swab, Human	FLZ, AMB, KTZ, ITC, VRZ	0.125	0.0625
C25	*C. krusei*	Urine, Human	CLT, TERB, KTZ, ITC	0.25	0.0625
C26	*C. krusei*	Onychomycosis, Human	FLZ, CLT, KTZ, ITC	0.25	0.125
C27	*C. albicans*	Onychomycosis, Human	KTZ, ITC, VRZ, MCZ	0.125	0.03125
C28	*C. krusei*	Urine, Human	CLT, AmB, KTZ, ITC, MCZ	0.0625	0.03125
C29	*C. krusei*	Calf, Diarrhea	AmB, TERB	0.25	0.0625
C30	*C. albicans*	Calf, Diarrhea	AmB, KTZ, MCZ	0.125	0.03125
C31	*C. albicans*	Calf, Diarrhea	KTZ, TERB, MCZ	0.25	0.0625
C32	*C. albicans*	Calf, Diarrhea	TERB, AMP, KTZ	0.25	0.0625
C33	*C. albicans*	Vaginal swab, Human	AmB, TERB, KTZ, ITC	0.0625	0.03125
C34	*C. krusei*	Sputum, Human	AmB, TERB, KTZ, ITC	0.25	0.125
C35	*C. krusei*	Urine, Human	FLZ, MCZ, AmB	0.125	0.0325

ITC: itraconazole, KTZ: ketoconazole, MCZ: miconazole, CLT: clotriconazole, FLZ: fluconazole, VRZ: voriconazole, TERB: terbinafine, CAS: caspofungin, and AmB: amphotericin B.

**Table 2:** *FKS*1 mutations in caspofungin-resistant *Candida* species.

Isolates No.	Species	Caspofungin MIC (µg/mL)	Caspofungin MIC after 0.5% Chitosan Treatment	Mutation (s) in *FKS*1	Amino Acid Change	Accession No.
C1	*C. albicans*	2	0.25	A1929T T1933C	S645P	PP663620
C2	*C. krusei*	4	0.5	C1934T	S645F	PP663621
C3	*C. krusei*	8	0.25	A1929T A1988G	E663G	PP663622
C4	*C. albicans*	2	1	T1922C	F641S	PP663623
C5	*C. krusei*	8	0.125	A1929T T2062A, A2064G	L688M	PP663624
C6	*C. albicans*	2	0.0625	C1934A	S645Y	PP663625
C7	*C. krusei*	2	0.25	T1933C	S645P	PP663626
C8	*C. albicans*	4	0.25	T1933C	S645P	PP663627
C9	*C. tropicalis*	8	0.125	G1932T	L644F	PP663628

## Data Availability

The datasets generated or analyzed during the current study are available from the corresponding author upon reasonable request.

## Supplementary Materials

The following supporting information can be downloaded at: https://www.mdpi.com/article/10.3390/antibiotics13070578/s1, Figure S1: Correlation matrix between different antifungals showing high positive correlation coefficients between the resistance to CLT and both KTZ and FLZ. High coefficients were detected between CAS and both CLT and VRZ; Figure S2: Antifungal potential of different chitosan concentrations against *Candida* species using agar well diffusion test. A high significant difference (*p* < 0.0001) was found between inhibition zone diameters of *Candida* species when tested with different concentrations of chitosan, with 0.5% being the most effective one. Table S1: Primers used in the study [16,52,64,65]; Table S2: Antifungal activity of chitosan (inhibition zone diameter mm) against *Candida species*; Table S3: Caspofungin inhibition zone diameters (mm) after treatment of *Candida species* with different concentrations of chitosan.
