# Supplementary material for: Fighting Emerging Caspofungin-Resistant Candida Species: Mitigating Fks1-Mediated Resistance and Enhancing Caspofungin Efficacy by Chitosan
Source: Antibiotics (Basel). 2024 Jun 22;13(7):578. doi: 10.3390/antibiotics13070578 (PMC11274059; doi:10.3390/antibiotics13070578)
Supplement: Supplementary file 1 [file antibiotics-13-00578-s001.zip › antibiotics-3047116-supplementary.pdf]

Table S1: Primers used in the study.

Gene	Primer	Reference
FKS1-RT	TGATACTGGTAATCATAGACCAAAAA<br>AACTCTGAATGGATTTGTAGAATAAGG	[63]
GCN5	TCATT AGATA GAACC GTTAG AGT<br>GAGAA GGCGA CAGAG TAG	[16]
ADA2	GTGATTGGGGAGCAGATGAA<br>CAGCCCAATTACCCAATCCA	[16]
ACT1	TGGACTTGTGTTGTTATCTGGACT<br>CTTGCTGTGTTTGTGTTTGTGTTG	[16]
FKS1- Sequencing		
Candida albicans	GAAATCGGCATATGCTGTGTC<br>AATGAACGACCAATGGAGAAG	[64]
Candida krusei	ACTGCATCGTTTGCTCCTCT<br>GAACATGATCAATTGCCAAC	[64]
Candida tropicalis	GGTTTAGATATGTGGATGTCCTA<br>AAATGTTTCTCCATGGAGTCAAG	[65]

References

63. Shivarathri, R; Tscherner, M; Zwolanek, F; Singh, NK; Chauhan, N;Kuchler, K. The fungal histone acetyl transferase *Gcn*5 controls virulence of the human pathogen *Candida albicans* through multiple pathways. *Scientific reports*. **2019**; 9:9445-50.

16. Shih, PY; Liao, YT; Tseng, YK; Deng, FS;Lin, CH. A Potential Antifungal Effect of Chitosan Against *Candida albicans* Is Mediated via the Inhibition of SAGA Complex Component Expression and the Subsequent Alteration of Cell Surface Integrity. *Front Microbiol*. **2019**;10:602.

64. Desnos-Ollivier, M; Bretagne, S; Raoux, D; Hoinard, D; Dromer, F;Dannaoui, E. Mutations in the fks1 gene in Candida albicans, C. tropicalis, and C. krusei correlate with elevated caspofungin MICs uncovered in AM3 medium using the method of the European Committee on Antibiotic Susceptibility Testing. *Antimicrob Agents Chemother*. **2008**; 52:3092-8.

65. Khan, Z; Ahmad, S; Mokaddas, E; Meis, JF; Joseph, L; Abdullah, A; Vayalil, S. Development of Echinocandin Resistance in *Candida tropicalis* following Short-Term Exposure to Caspofungin for Empiric Therapy. *Antimicrob Agents Chemother*. **2018**; 62: e01926-17.

Table S2: Antifungal activity of chitosan (inhibition zone diameter mm) against *Candida species*

Isolate No.	Chitosan concentrations			
	0.5%	0.25%	0.125%	0.0625%
C1	14	11	7	6
C2	12	10	13	8
C3	13	8	9	9
C4	14	10	8	10
C5	15	14	9	8
C6	13	13	8	7
C7	16	12	13	8
C8	12	9	10	9
C9	15	13	9	8
C10	15	8	14	12
C11	13	9	7	10
C12	15	14	8	9
C13	10	13	9	8
C14	12	12	10	0.5
C15	13	8	13	11
C16	14	10	10	No zone
C17	15	9	7	No zone
C18	13	10	8	7
C19	12	9	10	No zone
C20	11	8	9	6
C21	15	13	14	No zone
C22	10	7	9	No zone
C23	11	14	10	No zone
C24	11	8	8	No zone
C25	16	9	7	6
C26	17	10	9	8
C27	15	9	10	No zone
C28	16	12	11	10
C29	15	11	10	9
C30	12	13	10	9
C31	11	9	8	7
C32	11	8	9	7
C33	17	13	10	8
C34	15	9	8	7
C35	13	11	10	9

Table S3: Caspofungin inhibition zone diameters (mm) after treatment of *Candida species* with different concentrations of chitosan.

Isolate No.	Inhibition zone of caspofungin	Inhibition zone of caspofungin after chitosan treatment			
		0.5%	0.25%	0.125%	0.0625%
C1	No zone	11	12	14	13
C2	10	17	13	12	14
C3	0.9	20	16	19	13
C4	No zone	18	11	15	16
C5	10	20	18	14	15
C6	No zone	16	15	14	11
C7	0.8	14	13	12	11
C8	10	23	20	17	12
C9	11	14	12	0.8	10
C10	13	17	15	14	13
C11	13	14	12	18	16
C12	15	27	20	19	15
C13	15	16	15	14	12
C14	14	17	16	12	13
C15	18	14	12	13	15
C16	13	17	15	14	16
C17	19	25	21	19	15
C18	16	20	17	16	14
C19	18	25	20	16	13
C20	16	19	18	17	14
C21	17	19	15	15	12
C22	18	20	19	14	12
C23	20	23	20	19	17
C24	15	17	20	16	18
C25	17	20	15	11	No zone
C26	17	18	17	14	12
C27	16	21	19	15	15
C28	15	15	14	13	20
C29	13	17	14	13	13
C30	14	18	17	13	12
C31	14	20	19	18	16
C32	17	25	20	19	16
C33	18	20	18	15	12
C34	17	20	19	17	16
C35	16	21	19	18	16

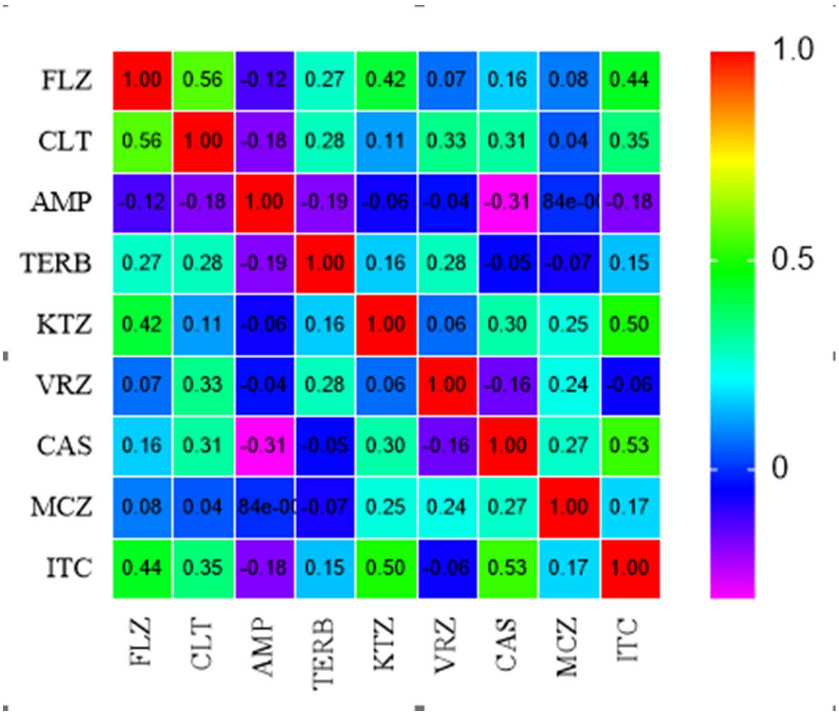

Figure S1: Correlation matrix between different antifungals showing high positive correlation coefficients between the resistance to CLT and both KTZ and FLZ. High coefficients were detected between CAS and both CLT and VRZ.

Chitosan 0.5	Chitosan 0.25	Chitosan 0.125	Chitosan 0.0625	*p*-value
13.485 ±1.27[a]	8.02 ±1.12[ab]	6.13 ±1.08[b]	3.26 ± 0.93[c]	<0.0001

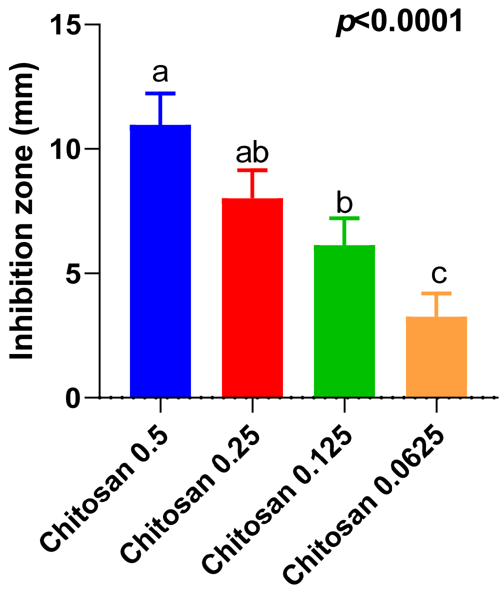

Figure S2: Antifungal potential of different chitosan concentrations against *Candida* species using agar well diffusion test. A high significant difference ($p < 0.0001$) was found between inhibition zone diameters of *Candida* species when tested with different concentrations of chitosan with 0.5% being the most effective one.
